# A Neural Network-Based Mesh Quality Indicator for Three-Dimensional Cylinder Modelling

**DOI:** 10.3390/e24091245

**Published:** 2022-09-04

**Authors:** Xinhai Chen, Zhichao Wang, Jie Liu, Chunye Gong, Yufei Pang

**Affiliations:** 1 Science and Technology on Parallel and Distributed Processing Laboratory, National University of Defense Technology, Changsha 410073, China; 2 Laboratory of Software Engineering for Complex System, National University of Defense Technology, Changsha 410073, China; 3 China Aerodynamics Research and Development Center, Mianyang 621000, China

**Keywords:** computational fluid dynamics (CFD), mesh quality, neural network, benchmark dataset

## Abstract

Evaluating mesh quality prior to performing the computational fluid dynamics (CFD) simulation is an essential step to ensure the acceptable accuracy of cylinder modelling. However, traditional mesh quality indicators are often insufficient since they only check geometric information on individual distorted elements. To yield more accurate results, the current evaluation process usually requires careful manual re-evaluation for quality properties such as mesh distribution and local refinement, which heavily increase the meshing overhead. In this paper, we introduce an efficient quality indicator for varisized cylinder meshes, consisting of a mesh pre-processing method and a neural network-based indicator, Mesh-Net. We also publish a cylinder mesh benchmark dataset. The proposed indicator is trained to study the role of CFD meshes on the accuracy of numerical simulations. It considers both the effect of element geometry (e.g., orthogonality) and quality properties (e.g., smoothness and distribution). Thereafter, the well-trained indicator is used as a black-box to predict the overall quality of the input mesh automatically. Experimental results demonstrate that the proposed indicator is accurate and can be applied in the mesh quality evaluation process without manual interactions.

## 1. Introduction

Computational fluid dynamics (CFD) plays a vital role in a broad spectrum of scientific and engineering fields, such as bioengineering, aerospace, energy engineering, and manufacturing [1,2,3]. During the CFD simulation, the quality of the generated mesh directly influences the solution accuracy and error magnitude. Many mesh generation methods have been proposed aiming to generate high-quality meshes [4,5]. Unfortunately, the quality of the initial mesh is usually not acceptable. The minimal mesh quality requirement is seldom achieved except on the most elementary problems [6,7,8]. Therefore, the procedure used to handle high-quality mesh generation is divided into three steps: initial mesh generation, mesh quality evaluation, and mesh optimisation. In this meshing process, an efficient mesh quality indicator is particularly important. The indicator determines the direction of subsequent quality optimization and ensures the accuracy of the desired solution. It serves as a basis for assessing the ability of generated mesh to faithfully represent the physics of the flow.

The high degree of complexity (non-linearity) between mesh quality and numerical accuracy makes the quality evaluation an extremely difficult task. It is hard to precisely define the relationship between mesh qualities and their correlations with numerical error [9]. Starting from the observation that regular or equilateral mesh elements are more pleasing, the traditional evaluation procedures focus on evaluating the shape of each element. Such a perspective leads to the formulation of quality indicators in terms of elemental geometric information such as area ratio, edge length, volume ratio, aspect ratio, skewness, minimum (maximum) angle, and gamma coefficient [10,11,12]. However, the above geometric-based indicators are often insufficient since: (1) They only yield geometric information on individual distorted mesh elements, and are useless for quality properties such as mesh distribution and local refinement. These properties affect the simulation accuracy in regions near boundary layers and wing-body configurations. (2) They may give inconsistent evaluation results for the same mesh (see [13,14] and references therein). Moreover, since geometric-based indicators may not guarantee an accurate result, the issue of mesh quality evaluation usually requires careful manual re-evaluation. This process relies heavily on the empirical, descriptive realm of a priori knowledge. As a result, the frequent human-computer interactions needed in the current evaluation process have become a bottleneck to the fully-automatic meshing process and significantly increase the meshing overhead. In order to ensure the cost-efficiency of meshing, it is essential to build an intelligent mesh quality indicator without manual interactions.

In recent years, artificial neural networks have been proven capable of learning complex mapping and replacing human labour in various applications [15,16,17]. The network utilises multiple layers of neural units to learn important features automatically from high-dimensional parameter spaces. By performing an optimisation procedure based on the loss function, the network model is able to approximate the complex and nonlinear mapping from training samples [18]. Despite the widespread success of neural networks in various physical problems, there have been only limited attempts at neural network-based mesh quality evaluation.

In this paper, we propose a mesh quality indicator for three-dimensional cylinders, resulting in a point-based mesh pre-processing method, a neural network Mesh-Net, and a cylinder mesh benchmark dataset. The proposed indicator takes mesh files as input and learns the potential correlations between the mesh quality and simulation error. Compared with traditional quality indicators, which focus on detecting distorted mesh elements, our indicator is more accurate. It considers both the effect of element geometry, such as orthogonality, and quality properties, such as smoothness and mesh distribution. Experimental results demonstrate that the well-trained indicator is able to predict the overall quality of cylinder meshes and achieves an accuracy of up to 98.05%. Moreover, it can be applied in the automatic mesh quality evaluation process without manual interactions, which significantly reduces the meshing overhead. We hope our work can provide future research directions that contribute to efficient mesh generation technology. The proposed benchmark dataset is publicly available at https://github.com/MeshDataset/3D-Cylinder (accessed on 4 October 2021).

The rest of the paper is organized as follows: Section 2 describes related works about existing mesh quality indicators. Section 3 gives details of the proposed neural network-based mesh quality indicator. The experimental results and discussion are presented in Section 4. The conclusion is finally outlined in Section 5.

## 2. Related Works

### 2.1. Traditional Mesh Quality Indicators

It is well known that poorly shaped meshes tend to slow convergence and cause instability during the CFD simulation [19,20]. In order to ensure the accuracy of the numerical solution, many indicators have been proposed to check the mesh quality before simulation.

Starting from the observation that regular or equilateral mesh elements are more pleasing, Strang and Fix [21] discussed the minimum angle condition of mesh elements. They stated that the smallest angle of mesh elements should be bounded away from zero. Berzins [6] supported this view and proved that elements with a relatively small included angle might have a negative effect on the solution of the linear algebra problem. Similar conclusions were proposed by Shewchuk [22], who showed that a prerequisite for high-quality mesh elements is that there should be no large included angles.

To make the conclusion more specific, Liu and Joe [23] proposed a quality indicator $Q_{L}$ to identify ‘sliver’ elements.

(1) $$Q_{L}=\frac{1}{8.48528V}\left[{\left(\sum \frac{l_{i}}{6}\right)}^{3}\right],$$

where *V* is the volume, and $l_{i}$ is the edge length of the examined mesh element.

Bank [24] presented a geometric indicator $Q_{B}$ for CFD mesh quality control:

(2) $$Q_{B}=\frac{\sqrt{3}}{12A}\left(\sum {l_{i}}^{2}\right),$$

where *A* is the area of the mesh element. Weatherill [25] introduced a similar mesh quality indicator in the evaluation process. It is defined by:

(3) $$Q_{W}=\frac{1}{3A}\left[{\left(\sum l_{i}\right)}^{2}\right],$$

Another indicator referred to as the *Scaled Jacobian Quality Indicator* was proposed in the CUBIT code for the mesh quality [5]. The *Scaled Jacobian* first computes the triple product at each node of the element corners using the other mesh nodes. It then computes the average of the corner Jacobians. The value of this indicator varies from minus one to plus one. A positive scaled Jacobian is usually considered the minimum quality for an acceptable computational mesh (called inversion-free). In contrast, the negative values of the *Scaled Jacobian* indicate the presence of distorted elements.

Quality indicators such as *Aspect Ratio*, *Diagonal Ratio*, *Edge Ratio*, and *Equiangle Skewness* are widely-used in CAE software as quality metrics for mesh elements [26]. For example, The *Diagonal Ratio* $Q_{DR}$ is represented as the maximum ratio of the element diagonals:

(4) $$Q_{DR}=\frac{max(d_{1},d_{1}\ldots d_{n})}{min(d_{1},d_{1}\ldots d_{n})},$$

where $d_{i}$ is the length of the element diagonal. By definition, the higher the metric value, the less regularly shaped the examined element. For equilateral elements (square quadrilateral elements or cubic hexahedra), the *Diagonal Ratio* $Q_{DR}$ is 1.

The above quality metrics provide shape specifications for mesh elements employing geometric formulas (the value usually ranges between 0 and 1, and 1 for an equilateral element). However, several sets of numerical results in [13] have demonstrated that employing different quality metrics to evaluate the same element may lead to inconsistent results. This conclusion is also confirmed by Gao et al. [14]. They performed a thorough numerical study to analyse widely-used quality indicators and their correlations with the stability and accuracy of the simulations. Nearly twenty quality indicators were tested on hexahedral elements. It was observed that the correlations among indicators are ambiguous. The derivation of some geometric element-based quality indicators applies only to specific applications.

Overall, present-day mesh quality indicators tend to assess geometric imperfections (shape, edge length, included angle, Jacobian) on mesh elements. Other considerations such as mesh density and distribution that ensure desirable simulation accuracy are ignored. This deficiency imposes a burden on careful manual re-evaluation, which significantly increases the meshing overhead [9,27].

### 2.2. Neural Networks for Mesh Quality Evaluation

In recent years, many researchers attempt to explore new methods for complex physical problems using artificial neural networks (ANNs). The main insight of ANNs is the capability of finding nonlinear approximations to complex functions based on the architecture of interconnected neurons. After suitable training, the ANNs are able to predict the desired output accurately. As a result, ANNs have been successfully applied to various CFD problems to improve efficiency and reduce overhead [9,18].

**Figure 1:**
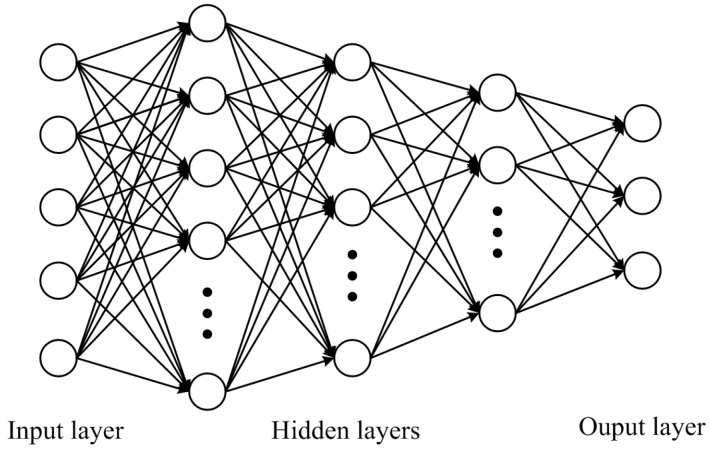
The architecture of MLP.

Multi-layer perceptron (MLP), which consists of several layers of neurons, is a specific type of ANNs [28]. The structure of an MLP is separated into three parts, the input layer, hidden layers, and output layer. A multi-layer perceptron with three hidden layers is shown in Figure 1. The neurons in the first hidden layer receives source signals from the input layer and propagate them to the succeeding layers. The signals are passed between all hidden layers (with activation functions) and finally converted into high-level features. The feature values in the output layer indicate the probability of the input belonging to a particular category. In this forward propagation process, the output of each layer is computed as:

(5) $$N\left(\vec{x}\right)=\sum_{i=1}^{m}\sigma \;\left[\sum_{j=1}^{n}\left(\omega_{ij}x_{j}+b_{i}\right)\right],$$

where *n* is the number of neural units in the hidden layer. *m* denotes the number of input units. ω is the weight, and *b* is the bias. The activation function is represented by σ. The loss function concludes the partial derivatives of the layer outputs with respect to the variables. After that, the adjustable variables in the neurons (weights and biases) are optimised via backpropagation to approximate the nonlinear mapping.

To better learn the local and contextual information from input data, convolutional neural networks (CNNs) are proposed for complex applications such as image classification, regression, and scene recognition [15,16,29]. CNNs employ shift-invariant filters (kernels) followed by pooling units to extract local and global features from feature maps. By minimising the loss function with many hyperparameters, the network obtains the optimum weights and biases for the solving problem.

Chen et al. [9,30,31] first introduced neural networks to the mesh quality evaluation task. They proposed an automatic quality indicator for 2D NACA0012 airfoil meshes using CNNs. The indicator takes geometric characteristics of each mesh element as input (the edge length x, edge length y, and maximum included angle), then feeds them into the construed neural network to identify poor-quality NACA0012 meshes. However, due to the geometric properties of the input features, the input constructing process is computationally expensive, and the proposed method is only applied to two-dimensional meshes.

In this paper, we propose a neural network-based mesh quality indicator, accompanied by a benchmark dataset for three-dimensional cylinder meshes. In the mesh pre-processing phase, the indicator first splits the cylinder mesh into mesh surfaces and extracts mesh points from each surface. The proposed neural network Mesh-Net directly takes mesh points as input without geometric calculation. During the training, Mesh-Net employs fully-convolutional and global average layers to learn the role of mesh geometry and distribution on the accuracy of CFD simulation. The well-designed architecture makes it attractive as an indicator of variable-sized three-dimensional cylinders. After suitable training, the indicator is able to predict the overall quality of the input mesh precisely. It can also be applied in the automatic mesh quality evaluation process without manual interactions.

## 3. A Neural Network-Based Mesh Quality Indicator for Three-Dimensional Cylinder Modelling

Neural network-based mesh training is the optimisation process by which the relation between the input mesh and quality prediction is established. This process usually requires a large number of labelled mesh samples to learn accurately. However, since annotating CFD meshes with simulation accuracy can be time-consuming and expensive, there has not yet emerged a public three-dimensional mesh dataset. To support our study and address the problem of available mesh datasets, we developed a cylinder mesh benchmark dataset for neural network-based mesh quality evaluation.

### 3.1. Three-Dimensional Cylinder Mesh Benchmark Dataset

In this section, we introduce the process of building the mesh benchmark dataset used for training. Each mesh sample generation can be divided into four steps: (1) modelling, (2) transforming, (3) simulation, and (4) annotation.

**Figure 2:**
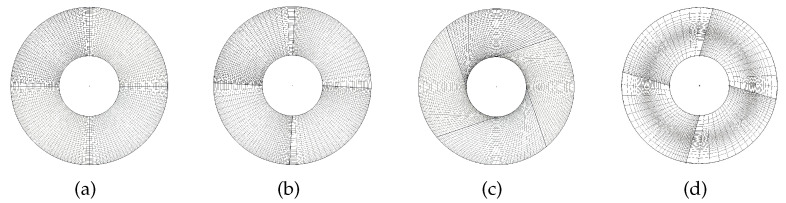
Mesh transforming used in the automatic three-dimensional cylinder mesh generator. (**a**) Initial mesh, (**b**) curve translation, (**c**) mesh surface rotation, (**d**) point reposition.

In the initial modelling step, the geometric model of the three-dimensional cylinder was constructed. Then, we generated meshes that varied in mesh size and deformed them to obtain cylinder meshes with different qualities. To this end, we developed an automatic three-dimensional cylinder mesh generator. The generator takes mesh files as input and transforms the input mesh using point reposition, curve translation, or mesh surface rotation. Figure 2 illustrates some of the deformed cases. We can see that a large degree of variance in geometric transformations can be achieved. Using this generator, we have collected a large dataset with 20,480 cylinder meshes that span different mesh sizes and contain a wide variety of quality properties. Notice that the obtained non-fixed size meshes increase the richness of the proposed dataset and make it useful for mesh training tasks involving multiscale cylinder models.

During the simulation step, we performed numerical simulations for each mesh sample on a classical problem. The problem models the steady laminar flow between rotating and stationary concentric cylinders (see material properties in Table 1) [32]. Considering the inner cylinder has radius $r_{0}$, angular velocity $w_{0}$, and temperature $T_{0}$, while the outer is $r_{1}$ and $T_{1}$, we calculate the tangential velocity in the annulus at certain radial locations. The motion equations include velocity component $u_{\theta }$, radius *r*, *T*, and *p* as:

(6) $$\frac{\partial u}{\partial \theta }=0$$

(7) $$\frac{dp}{dr}=\frac{\rho u_{\theta }^{2}}{r}$$

(8) $$\frac{d^{2}u_{\theta }}{dr^{2}}+\frac{d}{dr}\left(\frac{u_{\theta }}{r}\right)=0$$

(9) $$\frac{k}{d}\frac{d}{dr}\left(r\frac{dT}{dr}\right)+\mu {\left(\frac{du_{\theta }}{dr}-\frac{u_{\theta }}{r}\right)}^{2}=0$$

with boundary conditions:

(10) $$r=r_{0}:\;u_{\theta }=r_{0}\omega_{0}\;T=T_{0}\;p=p_{0}$$

(11) $$r=r_{1}:\;u_{\theta }=0\;T=T_{1}$$

After the simulation, we compared the numerical solution with target results in [32] at four radial locations (20 mm, 25 mm, 30 mm, and 35 mm), and manually divided each sample into one of the following four quality categories:

(1) High-quality Mesh: is a class of acceptable meshes with a very small error in the numerical solution.
(2) Non-orthogonal Mesh: occurs when the curves or surfaces of the mesh are not vertically orthogonal. Numerical experiments in [5] show that skewed mesh with poor orthogonality can affect the order of accuracy and error magnitude. Non-orthogonal meshes also have a negative impact on the convergence speed.
(3) Non-smoothness Mesh: is a class of meshes in which the length ratio is distorted, or elements are overlapped in complex domains. One approach to increase the quality is to smooth a collection of nodes (while preserving mesh connectivity) or to optimize node positions (vertex repositioning) [7,33].
(4) Poor-quality Mesh: represents meshes with poor orthogonality, smoothness, and distribution. According to the analysis in [34], poorly-shaped meshes can cause the ill-conditioned stiffness matrix problem and seriously affect the solutions of the partial differential equations.

**Figure 3:**
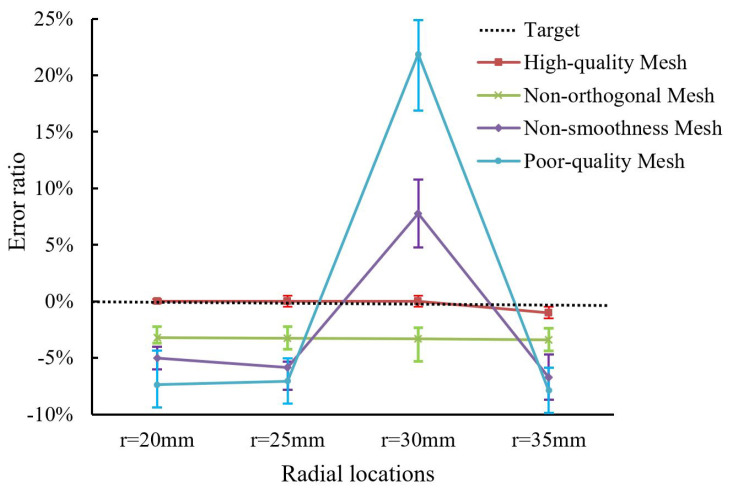
The numerical error of different quality meshes in the proposed benchmark dataset.

To verify the validity of the annotation procedure, we compared the numerical error of meshes in four quality categories. Figure 3 shows the numerical error of 20,480 meshes with different quality categories in the proposed benchmark dataset. We can learn that all high-quality meshes accurately simulate the fluid flow in the cylinder. For Non-orthogonal Mesh, there are small numerical errors (from −5% to 3%) during the CFD simulation. However, these meshes suffer from a slow convergence speed compared with meshes in the High-quality Mesh category. The numerical error of Non-smoothness Mesh ranges from 4% to 10%, while the poor-quality meshes leave a larger simulation error (up to 24.2%) compared to the target results.

**Figure 4:**
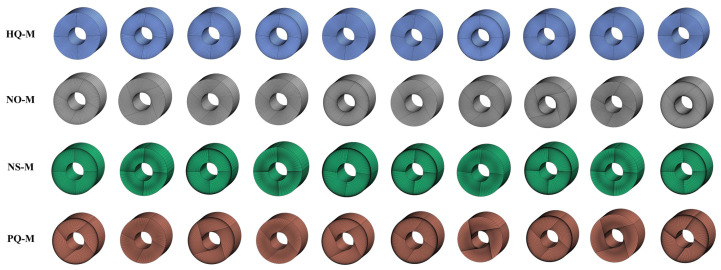
Mesh samples in the proposed mesh benchmark dataset. The dataset consists of 20,480 labelled cylinder meshes with different sizes and qualities. Each sample falls into one of four categories: High-Quality Mesh (HQ-M), Non-orthogonal Mesh (NO-M), Non-smoothness Mesh (NS-M), or Poor-quality Mesh (PQ-M).

Overall, we seek to construct a large collection of mesh samples with accurate solution-based labels. Such data is useful for supervised learning and neural network-based mesh quality evaluation. To achieve this, we built a three-dimensional cylinder mesh dataset containing a total of 20,480 meshes belonging to four categories, with an average of 512 meshes per size per category. The name and detail description of sizes and quality categories are listed in Table 2 and Table 3. Figure 4 shows some mesh samples in the proposed benchmark dataset. The diversity of the meshing ensures the richness and validity of the proposed dataset. We believe that this benchmark dataset contributes to developing advanced mesh understanding algorithms. It can also stimulate innovative research for CFD mesh quality evaluation tasks.

### 3.2. Mesh Pre-Processing

For the CNN-based mesh quality evaluation task, developing a representation scheme applicable to mesh samples is a prerequisite for neural network training. Due to the locally dense nature of CFD meshes, existing three-dimensional quantisation methods (e.g., multi-view or volumetric) do not apply to mesh samples. Point cloud features are able to handle the locally dense areas in underlying meshes. However, traditional point cloud representation ignores the spatial correlation between neighbouring points, which is crucially important for CFD mesh quality evaluation.

**Figure 5:**
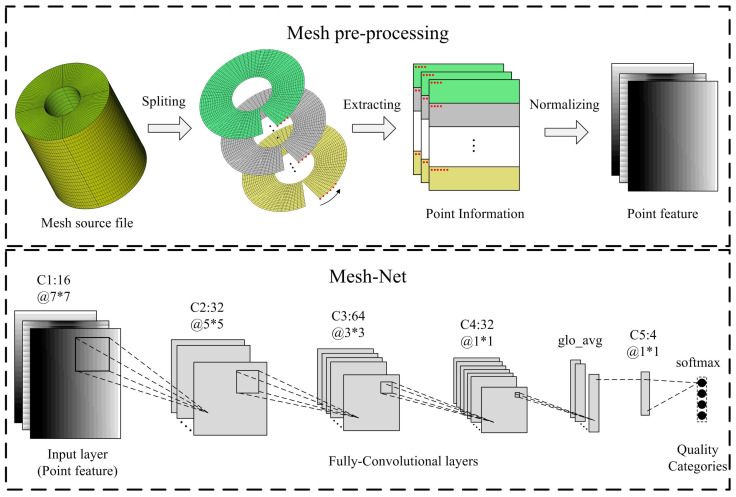
The proposed mesh pre-processing method and the architecture of Mesh-Net.

In our work, we introduce a point-based pre-processing method for cylinder mesh representation. In order to encode the spatial information of mesh points, we first split the cylinder into two-dimensional surfaces along the rotation axis, and then sequentially extract the mesh points from each mesh surface. After that, we combine the obtained point coordinates to form the three-channel point information matrix. Each channel of the matrix represents one of the dimensional coordinates (x, y, or z). The detail of the pre-processing method is shown in Figure 5.

Since the coordinates of each point are explicitly stored in the mesh source file, we can directly use the source file as training input. Compared to the mesh pre-processing in [9], which represents meshes using specific element features (edge length and included angle), our point-based representation is more efficient. It does not require any additional computation for three-dimensional cylinder meshes, which significantly reduces the pre-processing cost. Moreover, benefiting from the fact that the point information matrix incorporates spatial information, we can easily process input meshes without paying attention to the mesh size.

Normalisation is essentially a linear transformation that proportionally compresses and transforms a vector. This transformation keeps linear combinations and linear relational formulas intact, thus ensuring the robustness of a particular model. After normalising the input data, searching the optimal mapping in CNNs can be smoother (more likely to converge towards the optimal solution). Before training, we apply the standard deviation normalisation to the point information matrix. The normalisation formula is:

(12) $$x^{*}=\frac{x-\bar{x}}{\sigma_{x}}$$

(13) $$y^{*}=\frac{y-\bar{y}}{\sigma_{y}}$$

(14) $$z^{*}=\frac{z-\bar{z}}{\sigma_{z}}$$

where σ is the standard deviation of the original data, while $\bar{x}$, $\bar{y}$, $\bar{z}$ are the mean values of the original data, respectively.

Finally, the normalised feature matrix is fed into the proposed network Mesh-Net. The training process stops after converging to a local optimum. Thereafter, the trained network can be used as a black-box to analyse the meshing properties (smoothness, orthogonality, and distribution) from the cylinder point feature and automatically output the quality of the input mesh.

### 3.3. The Structure of Mesh-Net

We now describe the design of the proposed neural network Mesh-Net. It consists of an input layer, five convolutional layers, and a softmax layer. To keep the training and prediction cost low, we did not consider very deep architectures. The network architecture employs fully convolutional layers with no fully connected layer, which enables the network to take input meshes of arbitrary size and produce fixed size output. Figure 5 shows the architecture of Mesh-Net. As depicted in Figure 5, the number of channels (feature maps) in five convolutional layers is 16, 32, 64, 32, and 4, respectively.

As for the kernel size, we dynamically adjust the kernel size in each layer to obtain different receptive fields, rather than using the fixed kernels. At the beginning of the training, we prefer a relatively large receptive field to obtain more local point information. Inspired by the element dependency in the seven-point difference scheme, we set a 7 × 7 kernel in the first convolutional layer to capture the quality features of 49 adjacent mesh points. In the following layers, we gradually reduce the size of the convolutional kernel to obtain a smaller receptive field in high-level features. The kernel size of the next three layers is 5 × 5, 3 × 3, and 1 × 1, respectively.

There is no max-pooling layer in the proposed architecture. Instead, we set the stride in the first three convolutional layers to two to shrink the dimension of feature maps. It is worth noting that we employ a global average operation to calculate the mean value of the elements across dimensions in the fourth convolutional layers. After global averaging, the compressed feature maps are propagated to the softmax output function.

A loss function is employed during the network training phase to measure the discrepancy between the predicted output and the ground-truth tensor. In this work, we use *cross-entropy cost function* $L_{0}$ to measure the discrepancy between two probability tensors. $L_{0}$ is closely related to the Kullback–Leibler divergence, as given by:

(15) $$L_{0}=-\frac{1}{n}\sum \left[y\ln\hat{y}+\left(1-y\right)\ln\left(1-\hat{y}\right)\right]$$

where $\hat{y}$ represents the approximation of ground-truth *y*, and *n* is the number of samples in the mini-batch. Since each part of the network is differentiable, we can compute the derivatives of $L_{0}$ and update the parameters with respect to the input. The optimisation can then proceed via backpropagation (gradient descent). The weights of the network are updated iteratively by:

(16) $$\omega^{i+1}=\omega^{i}-\eta \frac{\partial L}{\omega^{i}}$$

where $\omega^{i}$ is the weight in the *i*-th forward propagation and η>0 is the learning rate. This process culminates with a vector-valued output that values in [0, 1]. It can be viewed as the neural network approximation of the desired function or the probability that the input mesh falls into one of four quality categories: High-quality Mesh, Non-orthogonal Mesh, Non-smoothness Mesh, and Poor-quality Mesh.

## 4. Experimental Results and Discussions

### 4.1. Training

As with any neural network, the choice of hyperparameters can strongly affect the prediction performance and the rate of convergence. In Section 3.3, we have determined hyperparameters, including the number of layers, the number of layer channels, and the kernel size. In addition, we need to define some training-related parameters, such as activation function in each layer, batch size, and learning rate.

The activation function used in Mesh-Net is responsible for introducing non-linearity into the network. Since the parameter update in each iteration involves the gradient of the activation function, the obtained tiny gradient can lead to a slow convergence or trapping in the local optimum [29]. To accelerate the convergence and avoid vanishing gradient issues, we equipped the convolutional layers with a composite function of the ReLU activation function and batch normalisation. Moreover, we use mini-batches to take a single training step and reshuffle the training set in each epoch after exhausting the entire training set. We find that the stochasticity introduced by shuffling improves the stability and performance in test cases.

The overfitting phenomenon is another problem existing in the training phase (i.e., the trained network ties too closely to the training set and behaves badly during testing). To tackle this problem, we combine the loss function with a regularisation term to avoid overfitting.

(17) $$L=L_{0}+\frac{\lambda }{2n}\sum {\left||\omega |\right|}^{2}$$

where $L_{0}$ is the loss function in Equation (15), ω represents the weights in Mesh-Net, *n* is the batch size, and λ is the regularisation coefficient.

**Figure 6:**
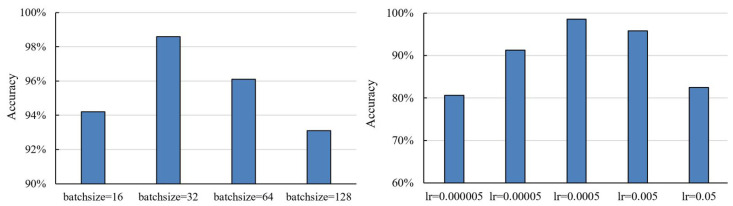
The performance of different batch sizes (**left**) and learning rates (**right**).

We use the Adam optimiser [35] with an exponential learning rate decay, which prevents the training from trapping in a local minimum. A comparison of different settings (batch size and learning rate) is shown in Figure 6. To make the best use of Mesh-Net, we set the initial learning rate to 0.0005, while the batch size was set to 32. The training was performed using the open-source machine learning library TensorFlow [36].

### 4.2. Prediction

To demonstrate the capability of Mesh-Net when used as a quality indicator, we compared the predictive power of different classifiers on three-dimensional benchmark datasets. During the experiment, we randomly shuffled the samples and employed the first 75% of meshes in each size for training and the latter 25% for testing. For each set, the proportion of different categories of meshes is equal. We ran the training 10 times and took the average accuracy as the final prediction result.

We compared the performance of the Mesh-Net with that of three widely-used machine learning algorithms and one multi-layer perceptron (MLP). The machine learning algorithms are support vector machine (SVM), quadratic discriminant analysis (QDA), and Gaussian Naive Bayes (GNB) [36]. The MLP used in this paper contains five layers, i.e., an input layer, three hidden layers, and an output layer. The number of neural units in three hidden layers is 16, 32, and 64, respectively. The number of neural units in the output layer is 4, which equals the number of quality categories.

Many metrics can be used to measure the performance of neural networks, such as recall, accuracy, and F1-score. Since the number of samples in each category balanced (512 × 10 per category), we only use accuracy to evaluate the performance of different classifiers. The accuracy acc is defined as:

(18) $$acc=\frac{TP+TN}{P+N}$$

where true positive (TP) is the number of correctly classified positive instances, true negative (TN) is the number of correctly classified negative instances, and P+N represents the total number of instances.

Table 4 reports the accuracy of different methods on the three-dimensional cylinder mesh dataset. Constrained by the classifiers’ limitations on the dimensionality of the input samples, we divided the experiments into two parts. The first part is the mesh training on fixed-size samples (Size 1 test). In this part, all five classifiers are trained and tested. In the second part, Mesh-Net, which accepts meshes of arbitrary size, is trained to test the overall performance across different mesh sizes (Full-size test). As can be seen in Table 4, the accuracy rate of machine learning algorithms is relatively low on the fixed-size test. All machine learning classifiers, SVM, QDA, and GNB, show a prediction accuracy of less than 90%. MLP achieves an accuracy of 95.70% on the mesh quality evaluation task. However, it is clear that the proposed CNN-based indicator is more effective than widely-used machine learning algorithms and MLP. It outperforms other trained classifiers and achieves an accuracy of 98.05% on fixed-size meshes and 96.60% on non-fixed size meshes.

**Figure 7:**
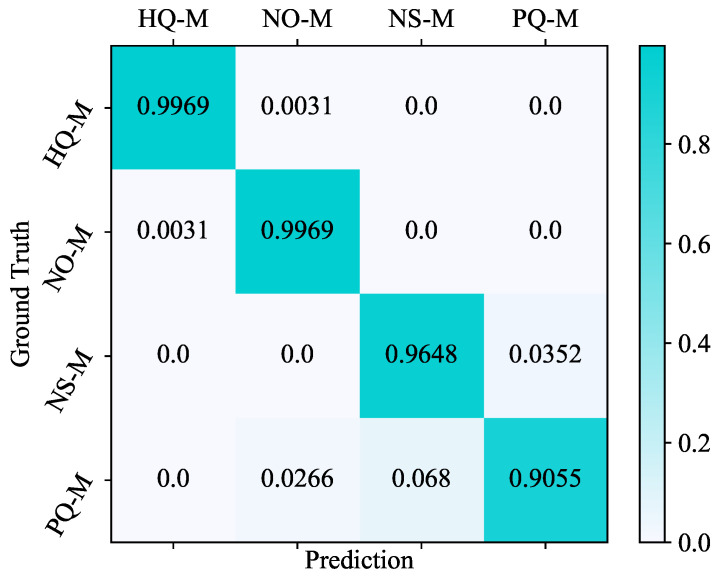
The confusion matrix of the full-size test.

To better understand the prediction across different categories, we present the confusion matrix of the full-size test (see Figure 7). We found that meshes with high-quality (HQ-M) and non-orthogonality (NO-M) achieve accurate quality prediction. Only four meshes (0.31%) were wrongly predicted. For meshes with non-smoothness (NS-M), 45 (3.52%) meshes were misclassified to poor-quality mesh (PQ-M). The category of PQ-M was predicted with the lowest accuracy. The results show that 121 (9.45%) poor-quality testing meshes were wrongly classified. Thirty-four (2.66%) PQ-M samples were misclassified to NO-M, and 87 (6.8%) PQ-M samples to NS-M. The inaccuracy is mainly because mesh quality properties such as non-orthogonality and non-smoothness are easily confused, especially when the point reposition or surface rotation happens. However, the incorrect predictions from Mesh-Net still make sense to the meshing procedure. It identifies part of the quality defects in the input mesh and guides the subsequent mesh optimisation.

We note that the computational complexity increases considerably with the number of neural units. The structure of the CNN-based indicator must be intelligent enough to make the classification task possible and simple enough to keep the training and prediction cost low. Thus, we did not consider very deep architectures. Moreover, the proposed network employs full convolutional layers without fully connected layers, which greatly reduces the number of parameters (orders of magnitude) compared with MLP. The introduction of fully convolutional layers also allows the input of meshes with different sizes.

Overall, we propose a CNN-based quality indicator for three-dimensional cylinder meshes. The proposed network Mesh-Net fully exploits the advantages of receptive field properties of convolutional neural networks. It employs different sizes of kernels to capture the local and global quality features of the preprocessed mesh. During the training, the network learns the relationship between the quality of the cylinder mesh and the error convergence of CFD simulation. Thereafter, the trained indicator can be used as an intelligent quality control model to evaluate mesh quality before CFD simulations.

## 5. Conclusions

Mesh-based methods have proved extremely useful in computational fluid dynamics simulations. During the cylinder simulation, the quality of the preassigned mesh affects the accuracy of numerical solutions. Poorly shaped meshes tend to slow convergence or cause analysing instability. Many quality indicators have been proposed to serve as quality control by analysing the geometric information of mesh elements. However, these element-based indicators do not necessarily provide reliable guidance for the subsequent optimisation process. They also require frequent human-computer interactions during the evaluation, which significantly increases the meshing overhead. Therefore, it is desirable to develop an intelligent indicator that automatically learns the quality of the mesh.

In this paper, we present an efficient mesh quality indicator by using convolution neural networks (CNNs). To support our study, we also release a three-dimensional cylinder mesh dataset, which contains 20,480 meshes, with different sizes and qualities. The proposed indicator is trained offline and employs a feedforward approximation to learn the mesh quality properties, such as orthogonality, smoothness, and mesh distribution. It takes mesh files as input and outputs the overall quality of the input mesh to determine if it meets the solver’s requirements. Experimental results show that the proposed method is accurate, computationally efficient, and straightforward.

We believe that the applications of deep learning methods to mesh quality problems are expected to address the challenges posed by frequent manual interactions and reduce the meshing cost. We also hope that the release of large-scale datasets can stimulate innovative research on mesh quality evaluation and advance the development of fully automatic mesh generation.

## Figures and Tables

**Table 1:** The material properties of the three-dimensional cylinder.

Parameter	Value
Density	1 kg/m${}^{3}$
Viscosity	0.0002 kg/m${}^{-5}$
Radius of the inner cylinder	17.8 mm
Radius of the outer cylinder	46.28 mm
Angular velocity of the inner wall	1 rad/s

**Table 2:** Ten different sizes of samples in the proposed mesh dataset.

Case	Mesh Size	Number of Meshes
Size 1	96 × 30 × 20	2048
Size 2	100 × 30 × 20	2048
Size 3	104 × 30 × 20	2048
Size 4	108 × 30 × 20	2048
Size 5	112 × 31 × 20	2048
Size 6	116 × 31 × 20	2048
Size 7	120 × 31 × 20	2048
Size 8	124 × 31 × 20	2048
Size 9	128 × 32 × 21	2048
Size 10	132 × 32 × 21	2048

**Table 3:** The name and detail description of four quality categories.

Label	Quality Categories	Number of Meshes
1 (HQ-M)	High-quality Mesh	512 × 10
2 (NO-M)	Non-orthogonal Mesh	512 × 10
3 (NS-M)	Non-smoothness Mesh	512 × 10
4 (PQ-M)	Poor-quality Mesh	512 × 10

**Table 4:** The performance of different classifiers on the cylinder mesh dataset.

Case	Model	Accuracy (%)
Size 1 test	SVM	89.06%
	QDA	87.30%
	GNB	79.49%
	MLP	95.70%
	Mesh-Net	98.05%
Full-size test	Mesh-Net	96.60%

## Data Availability

Not applicable.
